# Supplementary material for: Whole exome data prioritization unveils the hidden weight of Mendelian causes of male infertility. A report from the first Italian cohort
Source: PLoS One. 2023 Aug 4;18(8):e0288336. doi: 10.1371/journal.pone.0288336 (PMC10403130; doi:10.1371/journal.pone.0288336)
Supplement: S1 Fig — (DOCX) [file pone.0288336.s001.docx]

**Supplemental Figure 1**

**S**yndromic and non-syndromic genes form a highly interconnected network.

Panel A) analysis of non-syndromic genes (mapped 155 out of 156 genes): 2 clusters of which one related to mitosis, meiosis, and cell cycle regulations (87, red) and the other of genes related to flagellum, cilium and acrosome development (68, light green).

Panel B) analysis of syndromic genes (mapped 133 out of 136 genes): 2 clusters of which one related to flagellum, cilium and acrosome development (60, light green), and the other to the hypothalamic – pituitary – gonadal (HPG) axis (73, red). Several connections between the clusters can also be appreciated (dashed lines).


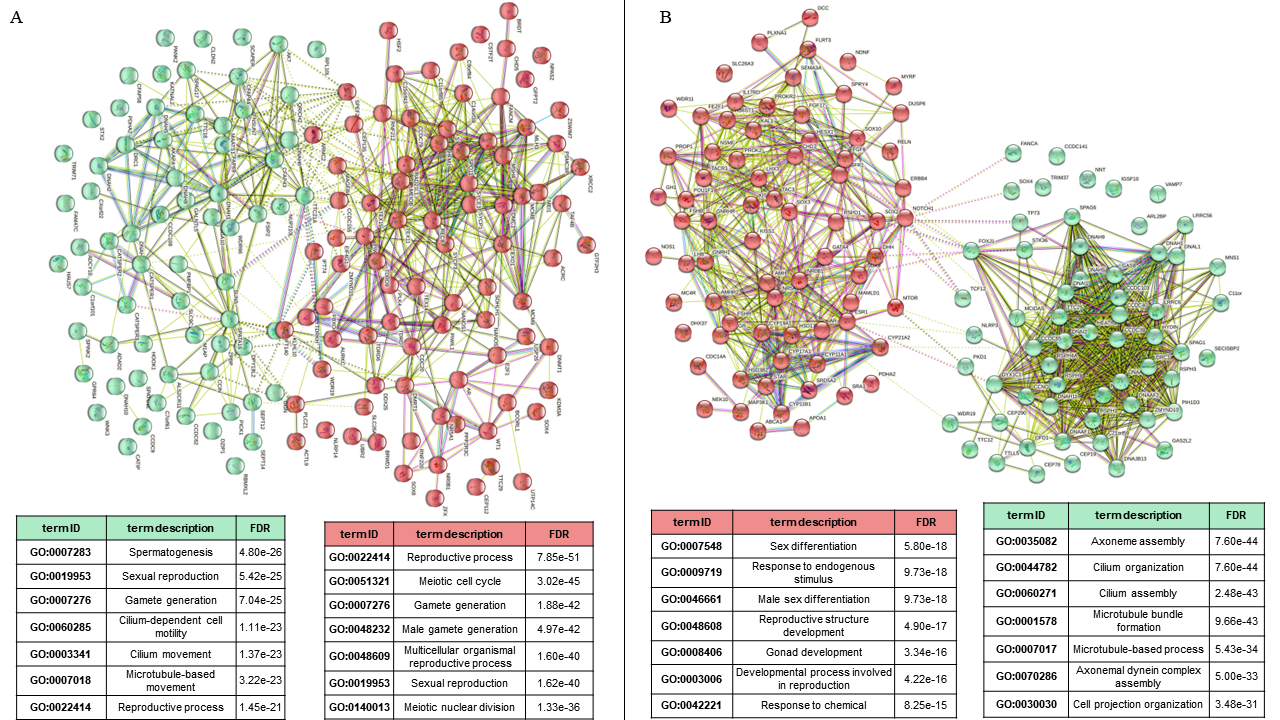


| **Table number** | **Description** |
| --- | --- |
| Supplementary Table I | ACMG criteria description |
| Supplementary Table III | biallelic LoF variant in the whole cohort |
| Supplementary Table III | INFERT_Lib genes |
| Supplementary Table IV | all ACMG classified variants selected with INFERT_Lib |
| Supplementary Table V | VUS variant in INFERT_Lib genes. O = patients with more than one VUS each; M = patients with one VUS each |
