# Supplementary material for: Whole exome data prioritization unveils the hidden weight of Mendelian causes of male infertility. A report from the first Italian cohort
Source: PLoS One. 2023 Aug 4;18(8):e0288336. doi: 10.1371/journal.pone.0288336 (PMC10403130; doi:10.1371/journal.pone.0288336)
Supplement: Attachment — Submitted filename: rebuttal to Reviewers.docx [file pone.0288336.s007.docx]

**Manuscript PONE-D-23-10435**

**Authors’ answers to Reviewers’ comments**

5. Review Comments to the Author

Please use the space provided to explain your answers to the questions above. You may also include additional comments for the author, including concerns about dual publication, research ethics, or publication ethics. (Please upload your review as an attachment if it exceeds 20,000 characters)

Reviewer #1: This study evaluates the unknown potential genetic causes in couples with pure male idiopathic infertility by applying variant prioritization to whole exome sequencing. This result is very interesting in that it provides a new understanding of male infertility etiology including Mendelian causes of infertility. I think that further understanding of the genetic impact on infertility need to improve treatment options of infertility.

AU_we thank the Reviewer for his comments.

Reviewer #2: The authors exam the gene of idiopathic infertile men. Even though the treatment is same, it's a new field for idiopathic infertile men, and maybe further become routine work up. Several gene is related to cancer in POI women. The author also mention some of the gene is also related to cancer in idiopathic infertile men.

AU_we thank the Reviewer for her/his comments.

Reviewer #3: The author of the paper entitled "Whole exome data prioritization unveils the hidden weight of Mendelian causes of male infertility. A report from the first Italian cohort" analyzed the whole exome sequencing of the males with idiopathic infertility. I have some comments:

1. only 12 patients of the studied cases diagnosed to have pathogenic and likely pathogenic variants. Please discuss by details (case by case) how you are sure that their infertility is because of the identified variants and also discuss the defect of this method (NGS) to diagnose this type of idiopathic infertility.

AU_We thank the Reviewer for the suggestions that will improve the readability of the manuscript. In the revised version we include a thorough and detailed description of patient phenotype, the genetic variant and the consistency with the mutant gene reported as associated to that specific form of infertility.

Also, we discuss about the consistency of NGS data vs. Sanger sequencing by referring to our experience as Clinical Genomics ward in the hospital. At page 9 in the “Variants prioritization and classification” paragraph, we add “Since high coverage has been considered as a sufficient quality indicator [23], Sanger confirmation has not been performed. Our personal data based on internal procedure validation for more than 2000 genetic variants diagnosed through the NGS, first, followed by Sanger sequencing confirmation, revealed an excellent concordance of variant calling (PC, personal communication).”

2. Insert TESE outcome Column to Table 2 for all 12 patients and discuss the relationship of the identified variants to TESE outcome.

AU_ Table 2 now includes a new column describing TESE outcome for the 12 mutant patients. TESE results are discussed at page 15 of the manuscript.
